# Elephants in the neighborhood: patterns of crop-raiding by Asian elephants within a fragmented landscape of Eastern India

**DOI:** 10.7717/peerj.9399

**Published:** 2020-07-02

**Authors:** Dipanjan Naha, Suraj Kumar Dash, Abhisek Chettri, Akashdeep Roy, Sambandam Sathyakumar

**Affiliations:** Endangered Species Management, Wildlife Institute of India, Dehradun, Uttarakhand, India

**Keywords:** Conflict, Local communities, Conservation, Demography, Mitigation, Himalayan foot hills, Agriculture, Tea gardens, Forest, Asia

## Abstract

Loss of forest cover, rise in human populations and fragmentation of habitats leads to decline in biodiversity and extinction of large mammals globally. Elephants, being the largest of terrestrial mammals, symbolize global conservation programs and co-occur with humans within multiple-use landscapes of Asia and Africa. Within such shared landscapes, poaching, habitat loss and extent of human–elephant conflicts (HEC) affect survival and conservation of elephants. HEC are severe in South Asia with increasing attacks on humans, crop depredation and property damage. Such incidents reduce societal tolerance towards elephants and increase the risk of retaliation by local communities. We analyzed a 2-year dataset on crop depredation by Asian elephants (*N* = 380) events in North Bengal (eastern India). We also explored the effect of landscape, anthropogenic factors (area of forest, agriculture, distance to protected area, area of human settlements, riverine patches and human density) on the spatial occurrence of such incidents.Crop depredation showed a distinct nocturnal pattern (22.00–06:00) and majority of the incidents were recorded in the monsoon and post-monsoon seasons. Results of our spatial analysis suggest that crop depredation increased with an increase in the area of forest patches, agriculture, presence of riverine patches and human density. Probability of crop depredation further increased with decreasing distance from protected areas. Villages within 1.5 km of a forest patch were most affected. Crop raiding incidents suggest a deviation from the “high-risk high-gain male biased” foraging behavior and involved proportionately more mixed groups (57%) than lone bulls (43%). Demographic data suggest that mixed groups comprised an average of 23 individuals with adult and sub adult females, bulls and calves. Crop depredation and fatal elephant attacks on humans were spatially clustered with eastern, central and western parts of North Bengal identified as hotspots of HEC. Our results will help to prioritize mitigation measures such as prohibition of alcohol production within villages, improving condition of riverine patches, changing crop composition, fencing agriculture fields, implement early warning systems around protected areas and training local people on how to prevent conflicts.

## Introduction

Growth in human populations, expansion of agriculture, livestock farming and shared nature of habitats force large mammals to come in conflict with humans. Human-wildlife conflicts also lead to antagonistic relationships between local communities, wildlife managers and conservationists further aggravating the problem of biodiversity conservation (Daskin & Pringle, 2016; Tilman et al., 2017). Attacks on humans, depredation of crops and livestock, and damage to property pose significant threat to human livelihoods and safety. Periodic losses reduce societal tolerance of local communities and prompt retaliatory killings, leading to local extinctions with impact on the overall ecosystem (Dickman, 2010; Ogada, 2014). Elephants symbolize large mammal conservation programs and are regarded as landscape engineers in Asia and Africa (Coverdale et al., 2016; Sekar, Lee & Sukumar, 2017). They range across large areas for dietary, reproductive requirements and forage on a diverse variety of grasses, shrubs, tree leaves, roots and fruits (Sukumar, 2003; Whyte, 2012). Home range size vary based on the abundance and distribution of resources with 100–1,000 km^2^ for Asian elephant and 11–500 km^2^ for African elephant herds (Thomas, Holland & Minot, 2008; Alfred et al., 2012). With rising anthropogenic impacts on natural ecosystems, humans and elephants occur in close proximity thus increasing the likelihood of conflicts (Sukumar, 1989; Hoare & Du Toit, 1999; Estes et al., 2011; Liu et al., 2017). Human–elephant conflicts (HEC) are not uniform due to the dynamic nature of ecological and anthropological factors which influence such incidents (De Boer et al., 2013). Hence, it is important to improve our understanding of HEC to match the dynamic nature of such events.

The intensity of HEC differs widely in Africa and Asia alongside variation in environmental factors such as the distribution of natural resources, agricultural practices, seasonal climatic conditions and socio-economic cultural beliefs (Shaffer et al., 2019). Fatal confrontations are relatively rare in Africa, yet increasing in developing regions of Asia (Mumby & Plotnik, 2018). Crop depredation is the most commonly reported form of damage, yet a rise in human injuries and deaths reduce social tolerance towards elephants in Asia and Africa (Sitati et al., 2003; Lenin & Sukumar, 2011; Lamichhane et al., 2018; Van de Water & Matteson, 2018). Small scale subsistence farmers are most vulnerable to damage by elephant attacks, crop raids (Riddle et al., 2010) and as a consequence, such low income groups engage in retaliatory killings, help organized poachers or prevent wildlife tourism based activities (Mackenzie & Ahabyona, 2012; Benjaminsen et al., 2013).

Asian elephants occupy only 5% of their historic range as a consequence of loss of forest cover and severe anthropogenic impacts on their habitats (Leimgruber et al., 2003). Only 22% of the current Asian elephant habitat is protected and the remaining is a matrix of multiple-use reserve forests, heterogeneous landscapes, crop fields, and human settlements. India has 60% of the global Asian elephant population while the rest are shared between Nepal, Myanmar, Thailand, Sri Lanka, Malaysia, and Indonesia (Sukumar, 2006; Fernando & Pastorini, 2011). An estimated 600 humans and 300 elephants die annually in India and Srilanka as a consequence of HEC with additional 600,000 families and 1 million hectares of land affected through crop raiding (Fernando et al., 2008; Pokharel, Singh & Sukumar, 2018).

Crop depredation is regarded as the stimulus of HEC (Webber et al., 2011; Mumby & Plotnik, 2018). Thus understanding how, when and where crop raiding occurs help wildlife managers focus on conflict hotspots, safeguard human livelihoods and implement appropriate mitigation measures. Spatial patterns of HEC are somehow positively related to human usage and the presence of settlements, agricultural fields in India, Nepal (Sukumar, 1991; Gubbi, 2012; Acharya et al., 2016), Thailand (Chen et al., 2016; Van de Water & Matteson, 2018) and Africa (Hoare & Du Toit, 1999). Conflicts are usually crepuscular and nocturnal with peaks during dusk and dawn (Venkataraman et al., 2005). Crop raiding is generally seasonal and occurs within the periphery of protected areas (Parker & Osborn, 2001; Chiyo & Cochrane, 2005). Mean annual rainfall which is considered as a surrogate of primary productivity was found to be positive with HEC in South-east Asia (Webber et al., 2011).

Crop raiding is a high-risk foraging behavior demonstrated by elephants especially males. To get easy nutrition, males undertake such risks when raiding crop fields and combined with their large ranging patterns are more likely to get involved in conflicts with humans compared to females (Pokharel, Singh & Sukumar, 2018). Crop raids can lead to retaliatory killings of elephants by local communities (Sukumar & Gadgil, 1988; Gubbi et al., 2014). Body size hypothesis predicts sexual segregation in bull and cow movement patterns in response to differential nutrient requirements with bulls preferring bulky diets over the quality of vegetation. Such a “high risk, high gain” strategy is often adopted by sub-adult, adult males to increase in body size and enhance reproductive success (Sukumar & Gadgil, 1988; Hoare & Du Toit, 1999; Rode et al., 2006; Chiyo et al., 2011). However, female elephants when in large groups also cause significant damage to subsistence farmers and commercial agricultural farms (Sitati & Walpole, 2006; Sukumar, 2006). Conflict occurs around the year, with seasonal peaks often coinciding with harvesting time of agricultural crops (Sitati et al., 2003). Elephants show risk avoidance strategy by evading areas of human settlements during the day and thus raid crops mostly at night (Sukumar et al., 2003; Graham et al., 2009; Gunn et al., 2014).

North Bengal region situated at the foothills of Eastern Himalaya, India is well known for the severity of human-wildlife conflicts with nearly five-hundred fatal attacks on humans by elephants (Naha et al., 2019) in the last 15 years. Almost twelve to thirteen percent of HEC cases in India occurs within this landscape. The region is highly fragmented with protected areas interspersed with tea plantations, crop fields (Naha, Sathyakumar & Rawat, 2018) and an increase in area of human settlements in the last decade (Naha et al., 2019). Human drunkenness is a major driver of HEC, with tea estate workers and farmers being the primary victims of fatal elephant attacks. Intoxicated people chase/harass elephants near settlements, crop fields and are attacked (findings from Naha et al., 2019). Rice beer (alcohol production) is also frequent within some of these villages and elephants are reported to visit such areas and damage crop, property. As a consequence, an annual sum of USD 67,479 and USD 78,930 was paid by the state forest department for compensating human casualty and crop damage to elephants respectively (Naha et al., 2019). Fatal elephant attacks were documented to be nocturnal with peaks during the monsoon season. The combined threat of a large number of fatal elephant attacks on humans and extent of crop raiding impose a substantial financial burden on wildlife authorities and a serious conservation problem for managing elephants. Although attacks on humans have been recently studied, lack of information on crop raiding remains a serious knowledge gap for mitigation of HEC within this region. It is needless to emphasize that a thorough understanding of crop raiding behavior would help to develop and direct appropriate mitigation measures and reduce the present extent of HEC.

Thus, through this present study, we investigate the spatial and temporal patterns of crops raids within a hotspot of HEC in South Asia. We also explore the effects of ecological attributes (tea plantations, agriculture, forest, distance from protected areas, length and extent of riverine patches), anthropogenic variables (human density, human settlements, length of roads) on the risk of crop-raiding by Asian elephants in North Bengal, eastern India. We (1) analyze the temporal and seasonal patterns of crop-raiding, (2) identify the spatial drivers and potential hotspots of crop-raiding, and (3) understand sex-biased crop-raiding behavior. Based on the review of previous studies on elephant activity (Sukumar et al., 2003; Graham et al., 2009) which suggests nocturnal patterns, we hypothesize that a higher number of crop-raiding events will occur during the night. Considering elephants to be a landscape dependent species (Hoare & Du Toit, 1999; Thomas, Holland & Minot, 2012; Bi et al., 2016), we hypothesize that probability of crop-raiding should be higher in areas with forests (refuge), periphery of protected areas and availability of water. Further, considering the “high-risk foraging behavior” which suggests that crop raiding is sex-biased (Chiyo & Cochrane, 2005), we hypothesize that the majority of crop-raiding incidents will involve lone bulls. Studies on HEC suggest spatial predictability in crop raiding (Ahearn et al., 2001) and our findings will aid in identifying potential crop depredation hotspots within the North Bengal landscape.

## Material & Methods

### Study area

**Figure 1:**
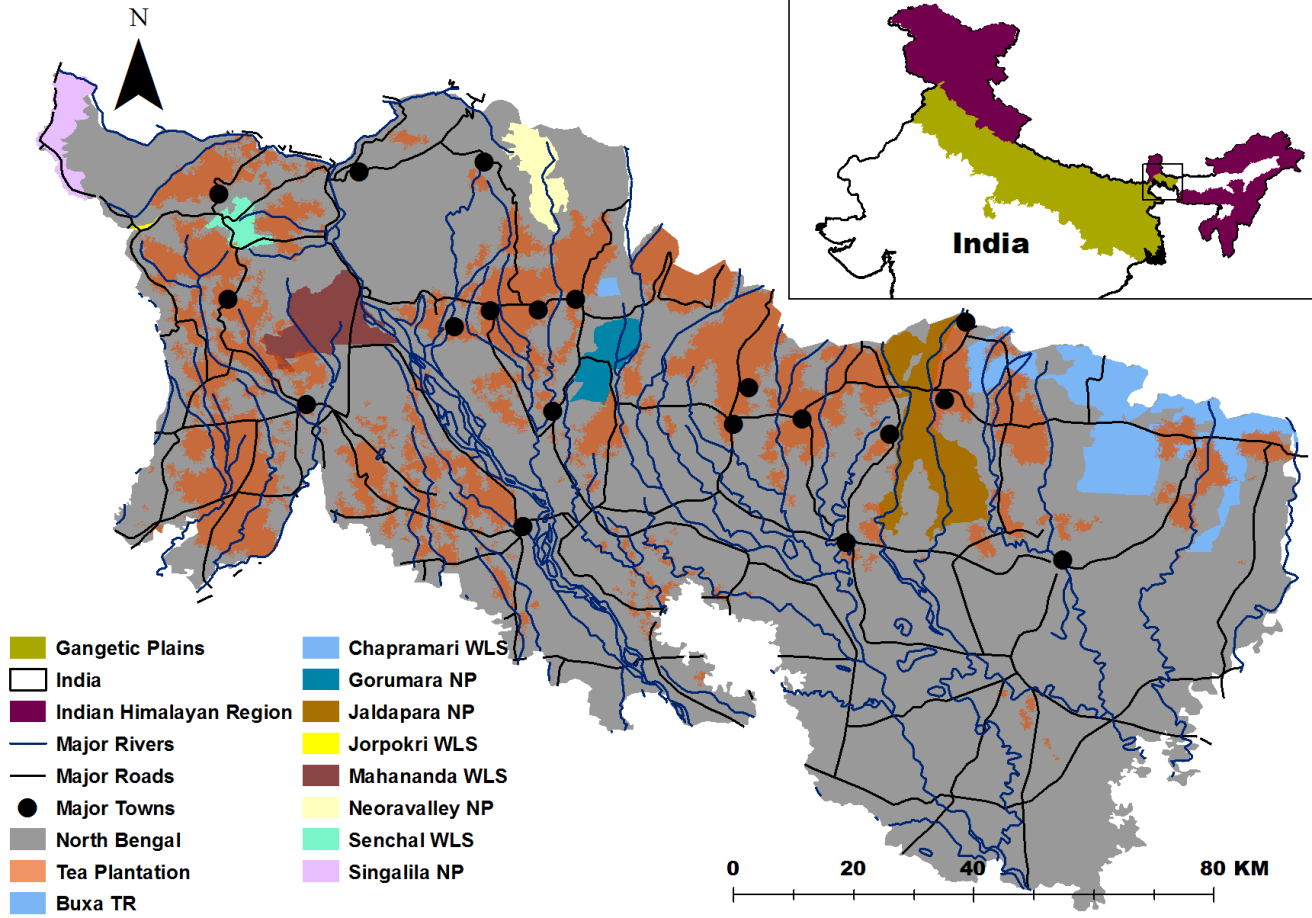
Study Area with the distribution of protected areas, rivers and towns.

The study site is spread across 5 districts of North Bengal (West Bengal state), eastern India (Darjeeling, Kalimpong, Jalpaiguri, Alipurduar, and Coochbehar) and encompasses an area of 12,700 km^2^ (Fig. 1). According to the bio-geographic classification of India by Rodger, Panwar & Mathur (2000), the study area falls under the two biogeographic zones i.e., the Himalaya and the Gangetic plains. This landscape is also known as Dooars, comprising of alluvial flood plains and intersected by several rivers draining into the Ganga—Brahmaputra delta in Bangladesh. A total of 3 National Parks (NP) i.e., Buxa Tiger Reserve and NP (761 km^2^), Jaldapara NP (220 km^2^), Gorumara NP (80 km^2^) and 2 Wildlife Sanctuaries (WS) i.e., Chapramari WS and Mahananda WS having an area of 9.5 and 158 km^2^, respectively are located in the foothills of the Dooars landscape. Neora Valley NP (88 km^2^), Singalila NP (78.6 km^2^) and Senchal WS (38.6 km^2^), Jorepokhri WS are located above 1,000 m altitude in the mountains. North Bengal historically was part of an extensive stretch of terai, alluvial grassland dominated forest extending from Nepal (mechi river in the west) to Assam (north eastern India, sankosh river in the east). Connectivity between the protected areas is poor with the landscape being highly fragmented by tea gardens, villages and urban settlements. The forest types are moist tropical, sub-tropical forests at the foothill region with major endangered large mammals being the Asian elephant (Elephus maximus), one horned rhinoceros (Rhinoceros unicornis), gaur (Bos gauras) and common leopard (Panthera pardus). Elephant population is estimated to be around 500 individuals spread across an area of 2,000 km^2^ (MoEF & CC Report , 2017).

According to the Human Census Data (Census Organization of India, 2020a,  Census Organization of India, 2020b, Census Organization of India, 2020c), an average range of (300–700) persons per km^2^ inhabit this region with a total population of 8.5 million. Primary occupation of local communities is agriculture, livestock rearing, and daily wage worker (tea estate). Major crops grown are paddy, jute, potato, maize and mustard with paddy cultivation carried out throughout the year. There are three varieties of paddy grown in the region viz., Aman, Aus and Boro with majority of the annual crop production (80%) derived from Aman and Aus. Harvesting period for this two varieties of paddy occur during monsoon i.e., July–August and winter i.e., November–December.

The livestock census 2012 reported a total of 3.5 million livestock in the region including cow, buffalo, goat, sheep, pig, and other with an average of 273 livestock per km^2^. Toto, Rava, Mech, and Bhutia are the major indigenous communities of the North Bengal region whereas the rest (Santhal, Oraon, Bhumij, Munda-Central Indian tribes) were either brought by the British planters or migrated from different regions of India to work in the tea gardens. This region eceives an annual rainfall of 3,160 mm with an altitudinal range of 50–3,500 m and the major seasons are summer (March–June), monsoon (July–October), and winter (November to February).

### Data collection

We analyzed data on crop-raiding by Asian elephants between January 2017 to December 2019. Our primary aim was to avoid strong spatial bias and hence we collected data (*N* = 380) locations from regions that were spatially spread out and not confined to specific localities within the landscape. We had informally constituted community-based village response teams (*N* = 25 teams with 5–7 members from each village) within the entire landscape and one primary task of such teams was to record and report incidents of HEC. To avoid exaggeration of losses (Siex & Struhsaker, 1999) we didn’t record data on the extent of crop damage. Once an incident was reported by the local community members, data collection was done by a team of researchers. Each researcher had a predefined area to be surveyed and a team of researchers allowed us to effectively sample the entire landscape. The research team recorded the GPS coordinates of the crop-raiding site, type of agricultural crops damaged, herd demographics, time spent during crop-raiding and time of raids (Appendix S1). Each crop-raiding incident was related to an occurrence of elephants within a particular locality (village) at a specific time. When our research team reached a particular village and elephants had left, data on the same parameters were collected through interviews with the local community members. There was also forest staff who were engaged by the local wildlife department to drive elephants from villages, crop fields and they also helped during data collection. These staff members visited the specific areas to confirm extent of damage and drove elephants from the crop fields. We verified the exact number of elephants involved within each event from the compensation records and also through direct communication with the staff members. The involvement of local community members, field researchers, and forest staff helped reduce bias and exaggeration of facts related to crop-raiding incidents. All field data were cross-checked at the Wildlife Institute of India, GIS lab and then imported to a geodatabase. The Government of West Bengal, Directorate of Forests gave the grant of permit (No. 5662/Wl/4R-6/2016) for this study.

### Conflict risk mapping

Data were analyzed as previously described in a study conducted on fatal elephant attacks on humans (Naha et al., 2019). We examined the seasonal and temporal patterns of crop depredation using the chi-square test (*α* = 0.05) (Zar, 2010) in R 3.4.0. We also examined difference in crops raided and human behavior, activity during crop raids using chi-square test in R 3.4.0. Monthly rainfall and crop damage frequencies (Pearce & Smith, 1998) were also explored using spearman correlations (Indian Ministry of Earth Sciences, 2020) in R 3.4.0. The study area was overlaid with 2,780 grids and 600 grids each with an area of 5 and 25 km^2^ respectively using Arc GIS 10.2.2. The cell size was selected as 5 km^2^ and 25 km^2^ based on an earlier study (Naha et al., 2019) to compare spatial patterns of crop damage and fatal elephant attacks on humans. We evaluated spatial autocorrelation among crop damage events within the cells (5 km^2^) using function moran.test (Moran’s I) in package (spdep) in R 3.4.0. We selected a total of 10 predictor variables based on their ecological importance to model HEC risk (Table S1). Land use data were categorized into 5 types (area of agriculture, forest, tea plantation, sand bed, riverine patches in m^2^). Distance from protected areas (m) was tabulated using the Euclidean distance tool for every grid. Data on anthropogenic variables such as length of roads (m), human density (per km^2^), and area of human settlements (km^2^) were extracted from the Digital Chart of the World (CIESIN, Columbia University), online human census data (Census Organization of India, 2020a,  Census Organization of India, 2020b, Census Organization of India, 2020c) and supervised vegetation map (Naha et al., 2019). We omitted slope, aspect and elevation, from the predictor variables since majority of the crop damage events occurred in flat lands. Our primary aim was to identify landscape predictors of HEC and hence we discarded distance to villages and considered area of human settlements (an artifact of human presence within rural, urban clusters) in a grid/cell (Pozo et al., 2017; Mukeka et al., 2019). After all predictor variables were compiled, they were extracted to the predefined grids and converted to raster files (ASCII format) using Arc GIS 10.2.2. The locations of crop raids were projected into UTM coordinates in Arc GIS 10.2.2 for all spatial analyses. The relationship between crop-raiding and the spatial variables was explored statistically using Arc GIS 10.2.2 and Maxent program. Maxent is an open access based species distribution program which is used to generate distribution of certain species/events based on a set of environmental/predictor variables (Phillips, Anderson & Schapire, 2006). A total of (*N* = 380) locations were used as sample data to run presence only species distribution models and model human–elephant crop depredation risk for the North Bengal landscape.

Maxent program calculated probability of conflict (crop depredation) based on the ecological predictors. Twenty-five percent of the locations were used as random test data or training to evaluate final model performance. We generated response curves for all individual variables and 204 jackknife estimator was used for computing final model output. We used 5 replicates to derive 205 model outputs with a total of 500 iterations. Accordingly, Maxent generated pseudo absence 206 points (10,000) from the entire study region (Elith & Leathwick, 2009). Details of the analytical procedure is provided as supporting information files (File S1).

## Results

### Seasonal and temporal pattern of crop-raiding

**Figure 2:**
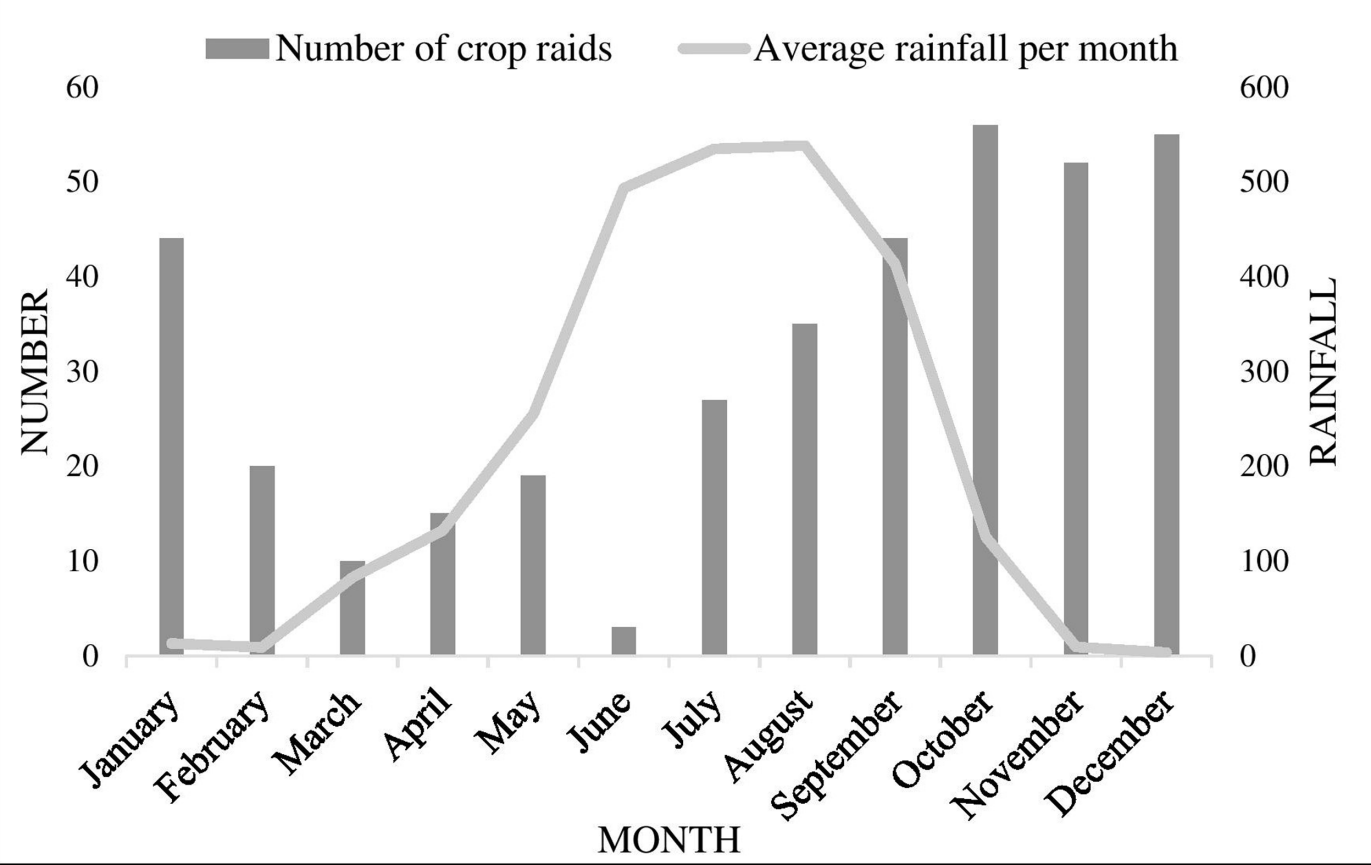
Graph displaying relationship between crop raiding frequencies by elephants and monthly rainfall in North Bengal. Bars denote number of crop depredation events by elephants and line denotes monthly rainfall in North Bengal.

In total, we recorded 380 crop-raiding incidents in the North Bengal region between 2017 to 2019. Crop-raiding events had major distinct peaks with 45% of the incidents recorded in winter between November to February, followed by 43% between July to October and rest twelve percent between March to June (*χ*^2^ = 19.86, *df* = 2, *p*-value < 0.05). Such crop raids coincided with harvesting of Aman and Aus varieties of paddy. There was a negative correlation between total number of crop raids and monthly rainfall (*r* =  − 0.306, *p* < 0.05) (Fig. 2). There was a distinctive nocturnal pattern with majority 89% of the incidents recorded between 10 PM–6 AM and the rest between 2 PM–10 PM (*χ*^2^ = 139.77, *df* = 2, *p*-value < 0.05). Majority of the crops raided were paddy (65%), maize (11%) and rest 25% comprised of seasonal vegetables, potatoes, cabbage, lentils, cauliflower, spinach, banana, jackfruit (*χ*^2^ = 45.42, *df* = 2, *p*-value < 0.05). Elephant crop raids occurred in flat areas with an average elevation of 117 m (SE 35).

### Demography of crop-raiding elephants

The mean group size was 23 SE 14.1 (range 2–150). Fifty-seven percent of the crop-raiding events involved mixed groups whereas 43% of the incidents involved lone bulls (sub-adult to adult males). Mixed groups composed of adult females, sub adult females, bulls and calves.

### Time spent in crop-raiding

Elephants spent an average of 308 min i.e., 5 h (SE 167 min) during crop-raiding range (15 min to 15 h).

### Human behavior and activity during crop-raiding

**Figure 3:**
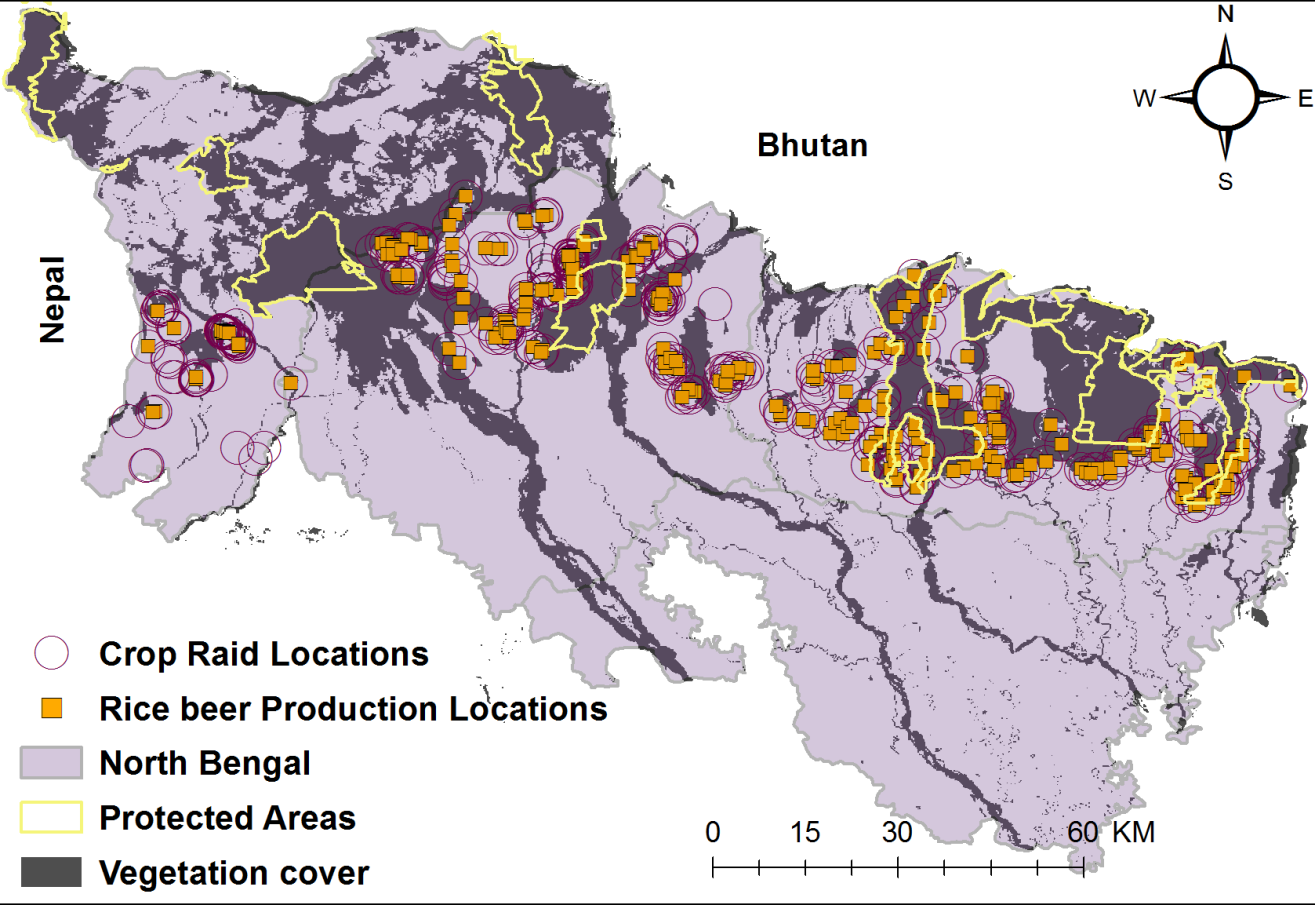
North Bengal landscape with the distribution of protected areas, rice beer production units and elephant crop depredation locations.

During crop-raiding, 61% of the people in the neighborhood were busy guarding agricultural fields, 30% were sleeping, 6% of the local community members were chasing the elephants whereas rest were engaged in household work (*χ*^2^ = 178.74, *df* = 2, *p*-value < 0.05). An average of 6 persons (range 1–20) were present in crop fields chasing elephants. From interviews with the local community members, we recorded that 75% of the localities raided by elephants had presence of locally brewed rice beer “haaria” production units’/storage chambers. Rice beer production units were concentrated around forest edges and periphery of protected areas (Fig. 3).

### Distance of crop depredation sites to nearest forest patches

We recorded that crop depredation sites were located within close proximity of forest patches. The average distance of a crop field raided by elephants was estimated to be 1.6 km (SE 1.5) (range 0–18.5 km) from the nearest forest patch. Thirty-five percent of the villages were located within 500 m of a forest patch whereas overall 63% of the incidents occurred within 1.5 km.

### Influence of landscape, anthropogenic variables on crop-raiding by elephants

Moran’s *I* identified spatial clusters of crop depredation within the North Bengal landscape. The *z* value (13.148), Moran’s Index (0.174) and (*p* value < 0.01) indicate that there was less than 1 percent likelihood that this pattern was due to random chance. The threshold distance was estimated to be 2,236.42 m. Maxent program used a total of 228 locations for training whereas 76 locations were used for testing. Based on this training and testing data set, final crop depredation risk maps and predictions were generated. A total of 5 replicates were used for model averaging and convergence.

Probability of crop depredation by elephants within a 5 km^2^ grid were best explained by a combination of ecological, anthropogenic attributes such as (i) area of riverine patches, (ii) area of agricultural fields, (iii) length of rivers, (iv) distance from protected areas, and (v) Human density. Receiver operating characteristic curve (ROC) value was estimated to be 0.89 (Fig. S1). Area of riverine patches which indicates availability of water within a grid was identified as the most important predictor of crop depredation.

Within a 5 km^2^ gird, crop raiding risk increased initially with an increment in area of agricultural fields (<5 km^2^) and then declined rapidly. Probability of crop raiding were highest in areas with water (>600 m^2^), forests (refuge), tea plantations (4,000 m^2^) and vicinity of protected areas (refuge). Anthropogenic variables such as human density (<40 persons/km^2^) and area of human settlements (<1,500 m^2^) were positively related to probability of crop depredation whereas such incidents decreased with increase in presence of roads (700 m) within a grid.

For 25 km^2^ grids, risk of crop damage increased with an increment in area of agricultural fields (>13,000 m^2^), tea plantations (>10,000 m^2^), forest patches (>20,000 m^2^) and human density (>42 persons/km^2^). Risk of crop raiding decreased with increase in distance from protected areas (>1 km), area of riverine patches (>6,000 m^2^), length of rivers and length of roads. Probability of crop depredation were best explained by a combination of ecological, anthropogenic variables such as (i) distance from protected areas, (ii) area of forest patches, (iii) area of tea plantation, (iv) area of riverine patches, (v) length of roads, (vi) area of human settlements, and (vii) area of agriculture fields. At a landscape scale, distance from protected areas was identified as the most important predictor of crop depredation. Receiver operating characteristic curve (ROC) value for the 25 km^2^ grid-based final model was estimated to be 0.83.

### Hotspots of conflict

**Figure 4:**
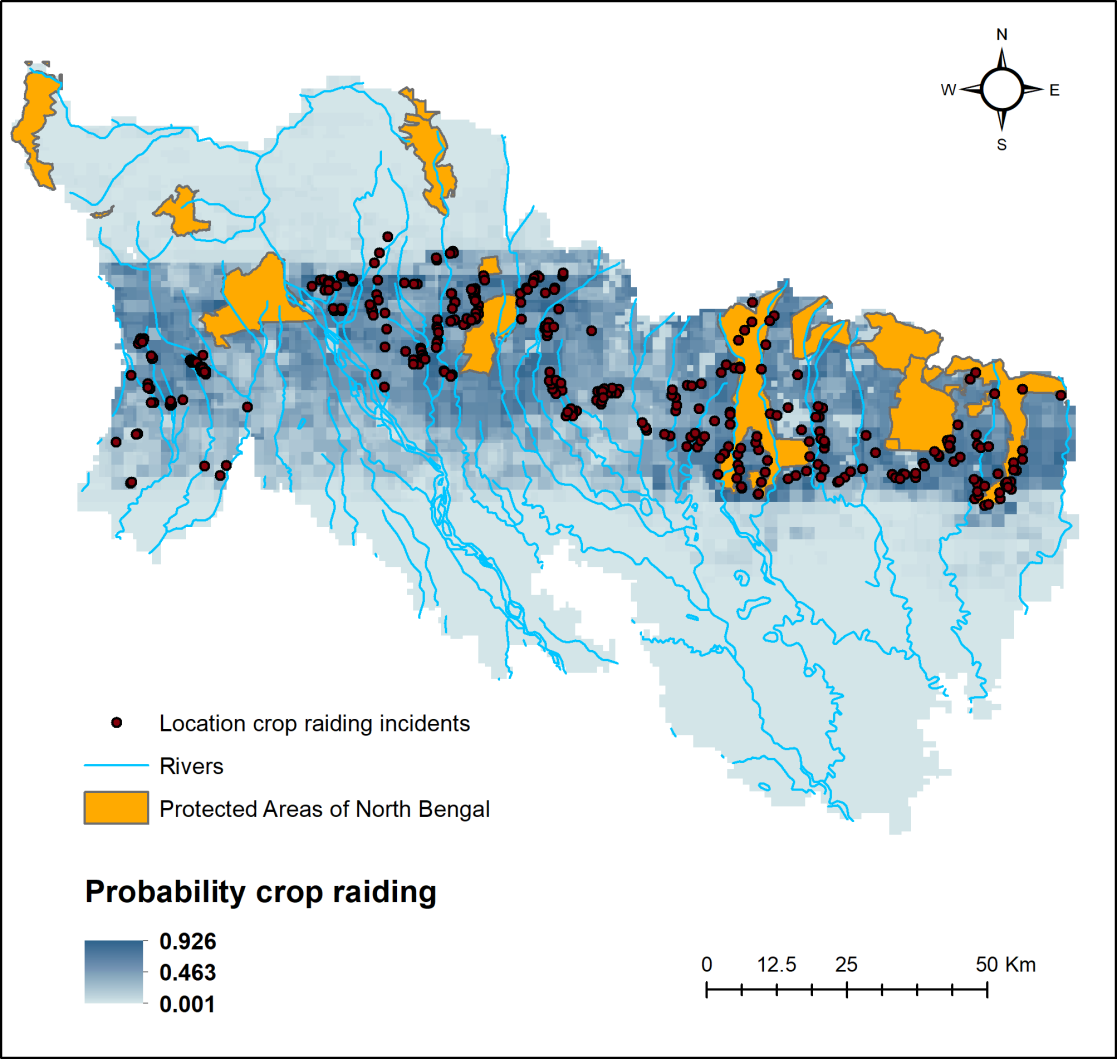
Hotspot of human-elephant conflicts with locations of crop depredation events.

The predictive maps based on the maxent models indicate eastern, central, and western parts of the North Bengal region as HEC hot spots (Fig. 4). Crop raiding probability increased near the periphery of protected areas (Mahananda WS, Gorumara NP, Jaldapara NP and Buxa NP), major forested corridors and the tea growing belt within the landscape.

## Discussion

Our analysis of crop-raiding data together with predictor variables generated new information on the potential drivers and spatial distribution of HEC in South Asia. Analysis of the temporal patterns supports the hypothesis that crop-raiding by elephants was nocturnal in nature which exhibits avoidance behavior of peak human activity. In line with our 2nd hypothesis, our model also confirms that elephants being a landscape dependent species, probability of crop-raiding are higher in areas with a matrix of agriculture, forests, riverine patches, tea plantations and periphery of protected areas. Contrary to our 3rd hypothesis, crop-raiding incidents involved both mixed groups and lone bulls.

Our results also suggest that the probability of crop-raiding increased with increasing human density (till a critical threshold of 40 persons/km^2^). Elephant raids peaked in areas located within a distance of 1,500 m from forested areas. Local community members proactively guarded their crop fields and chased elephants from the neighborhood. Villages located at the periphery of protected areas and forest refuge were the most affected by HEC. Attacks by Asian elephants on humans were recorded outside protected areas near human settlements and in the vicinity of crop fields in Nepal and India (Acharya et al., 2016; Naha et al., 2019). Results suggest seasonal variation in crop raids with eighty-eight percent of the incidents recorded in the monsoon and post monsoon seasons i.e., between July–February. Unlike in parts of South-east Asia where crops raids are positively correlated with monthly rainfall (Webber et al., 2011), we did not document any positive association of rainfall and crop raiding frequencies in the North Bengal region.

The most interesting finding of our study was that elephants raided villages where alcohol production (haaria-rice beer) was prevalent. Alcoholism (human drunkenness) is a major driver of fatal elephant attacks in this region and people intoxicated with rice beer have been reported to harass and chase elephants from villages, crop fields (Naha et al., 2019). As a consequence, more than five hundred people have been killed by elephants in the past 10 years (Naha et al., 2019). Similar patterns have been reported from Assam (India) and terai region of Nepal where HEC victims were drunk and chasing elephants (Lahkar et al., 2007; Lenin & Sukumar, 2011; Neupane, Johnson & Risch, 2013). Rice beer production is a community based activity and this alcoholic drink is produced from par boiled rice (paddy), ivy gourd and other locally available herbs. Once all raw ingredients are gathered, small tablets are prepared and dried in the sun. Dried tablets are kept within gunny bags for incubation which takes 2–6 days depending on the weather condition. Once the tablets are ready they are mixed with boiled rice, mixed with water and transferred to a fermenter within the village. The total incubation period for this preparation is 3–5 days and subsequently the fermented stock emits a strong pungent smell which attracts elephants (Ghosh & Das, 2004). Hence, such rice beer (alcohol) breweries should be relocated from the vicinity of villages to avoid frequent visitation by elephants and reduce the current extent of HEC.

Though spatial drivers of HEC are influenced by land-use patterns and anthropogenic factors, seasonality of such events are governed by the agriculture calendar. Seasonal patterns of crop raids coincide with monsoon and winter months when maize and paddy are ready to be harvested. Crop raiding has been widely documented to coincide with the harvesting pattern of major agricultural crops in Africa and Asia (Sitati et al., 2003; Chen et al., 2016). There are three varieties of paddy grown in this region i.e., Aman, Aus and Boro. Crop raiding has two distinct peaks which coincide with the harvesting of Aus and Aman varieties of paddy. Such patterns are similar to the adjoining Assam region where crop depredation occurred between August to December (Wilson et al., 2015). Female elephants are reported to be in peak sexual activity during monsoons which could be another major driver of crop-raiding peaks in monsoon months (Sukumar et al., 2003; Webber et al., 2011). Seasonal patterns of crop-raiding and fatal elephant attacks on humans also exhibit a similar trend with peaks during monsoon and winter months (Naha et al., 2019). Hence, we recommend intensification of mitigation measures during these two major crop raiding periods.

Data on the demography of crop-raiding elephants suggests that incidents involved an equal proportion of mixed groups and lone bulls. Our results are similar to findings from the neighboring region of Assam where crop-raiding involved smaller mixed groups comprising of adult females, sub-adult individuals and calves. The average herd size for crop-raiding elephants was 23 which is similar to the herd size of 18 elephants reported from the Assam region (Wilson et al., 2015). This foraging behavior is different from the male-biased crop-raiding behavior reported from other regions of South Asia and Africa (Sukumar, 1991; Graham et al., 2009; Goswami et al., 2015). Bulls, in general, are reported to use marginal habitats (Hoare & Du Toit, 1999) and crops constitute 10% of their overall diet as compared to 2% for herds (Sukumar et al., 2003). With the current loss of forest cover (>30%) in the region during the past few decades, elephants have been forced to rely on agricultural crops and the surrounding anthropogenic landscape for access to food and water (Lenin & Sukumar, 2011; Wilson et al., 2015). Unless the functionality, quality of existing elephant habitats, and dispersal corridors are revived, the present extent of crop-raiding and attacks on humans will increase (Lenin & Sukumar, 2011; Wilson et al., 2015). Appropriate mitigation measures such as restoring existing forest patches, increasing natural forage within protected areas and regulated crop cultivation should be the topmost conservation priority (Wilson et al., 2015).

Our results suggest that a matrix of landscape elements such as the area of agriculture, distribution of protected areas, availability of water and tea plantations are major drivers of HEC. North Bengal was once a contiguous elephant habitat extending from Nepal in the west to Myanmar in the east (Chowdhury et al., 1998). In recent times, the landscape has been severely fragmented with the construction of dams, linear infrastructure, human settlements apart from the presence of agriculture lands and tea plantations (Sukumar et al., 2003). Forest cover is primarily restricted to the protected areas, major wildlife corridors and reserved forests. Though there are numerous tea plantations in this region, they don’t provide forage and only act as temporary refuge for elephants (Chartier, Zimmermann & Ladle, 2011). Probability of crop raiding increased with area of agriculture fields within 25 km^2^ grids which was similar to findings of an earlier study on crop depredation by African elephants in Trans Mara area of Kenya (Sitati et al., 2003) and Asian elephants in north-eastern India (Wilson et al., 2015). Risk of human injuries and deaths to elephant attacks were also documented to be higher in such areas with presence of forest patches and agriculture fields (Naha et al., 2019). Thus, risk of crop raiding and human injuries, deaths were spatially clustered within specific land use types and such areas should be completely avoided by local communities during night.

The probability of crop-raiding was largely restricted to 1.5 km surrounding forested regions (refuge) and hence local communities residing within such areas were at the highest risk. Our results also highlight that crop raiding risk was highest within close proximity of protected areas and increased with human density. Local communities residing at the edge of forests, protected areas here are a combination of ethnic tribes (Rajbanshi, Mech, Rava, Gorkha, Tamang) and immigrants (tribes from central India such as Santhals, Oraon, Munda) who are either employed as tea estate workers or involved with subsistence agriculture. The major victims of elephant attacks are also such community members (tea estate workers and marginalized farmers) (Naha et al., 2019). Elephants are part of the local folklores and form an important part in the socio-cultural beliefs of tribal communities. Studies on HEC suggests that such incidents increase within close proximity to protected areas, forests (Nyhus & Sumianto, 2000; Difonzo, 2007; Lahkar et al., 2007; Riddle, 2007) and are generally confined within 1 km of the protected area (Sukumar, 1989). Previous studies in south, south-east Asia, and Africa have reported a loss of forest cover and rising human densities as major drivers of HEC (Leimgruber et al., 2003; Neupane, Johnson & Risch, 2013; Hoare, 2012). HEC show a positive relationship with human density, and research in Zimbabwe suggests that African elephants will adapt to humans till a critical threshold is reached which is 15–20 persons/km^2^ (Hoare & Du Toit, 1999). Our results also confirm that the probability of crop-raiding increases with human density and then decreases (threshold value 40 persons/km^2^) which is an artifact of elephant avoidance of dense human settlements. Human density in North Bengal was fairly high (range 200–700 persons/km^2^) and large settlements also act as barriers to elephant movement (Fernando et al., 2006). Majority of conflicts happen when they traverse such human used areas (Lenin & Sukumar, 2011). Mitigation measures should be focused on specific crop depredation zones within the landscape such as commercial agricultural farms and human settlements within close proximity of protected areas.

Our results confirm previous findings that HEC increases with an increment in crop fields. Studies on HEC in north-eastern India (Wilson et al., 2015) reported conflicts to be positively related to distribution of villages and refuge areas whereas in Kenya conflicts were positively related to the location of agricultural fields and their proximity to towns and roads (Sitati et al., 2003). Primary productivity has been identified as a major driver of HEC in Africa because dry arid savannahs are generally devoid of crops. The problem intensifies with an increase in crop production (Sitati et al., 2003; Wilson et al., 2015) such as in Asia where crop fields, human settlements provide food and forage, whereas forest patches, plantations act as day refuges within anthropogenic landscapes.

Distribution of water plays a major role in movement of large mammals within an ecosystem. Numerous studies in Asia and Africa have highlighted availability of water, swamps, streams and rivers as crucial drivers of habitat use by elephants within a landscape (Fernando et al., 2006; Duffy et al., 2011). Limited literature on Asian elephants suggests that forage, water (Sukumar, 1989) and anthropogenic impacts are significant predictors of resource use (Desai & Baskaran, 1996). Presence of water also influences the extent of a rice-based agricultural system, human settlements which further explains the importance of riverine patches as major spatial drivers of crop raids and fatal elephant attacks in North Bengal (Naha et al., 2019). Our results thus confirm that in a fragmented landscape, access and availability of water is a major spatial driver of HEC.

To safeguard elephants and humans within heterogeneous landscapes, multiple sociological factors should be addressed for developing successful conservation programs (Shaffer et al., 2019). Mitigation strategies should focus on keeping elephants out of crop fields and human settlements rather than confining them within fenced reserves. Elephants are dependent on forest patches, protected areas for movement, resting, forage and hence maintaining connectivity within such patches should be the topmost priority (Goswami & Vasudev, 2017). Forest patches in the vicinity of human settlements should be restored and encroachment of riverine patches should be minimized. There should be a prohibition on rice beer production and instead breweries should be relocated from the vicinity of villages to nearby urban centres. Breweries should be constructed with durable material to avoid any damage by elephants. The district administration should provide financial support/loan to the village communities to set up these breweries, shops/counters within the urban centres and commercialise production and sale of bottled traditional “North Bengal” rice beer. Such a program will provide local employment, generate revenue and reduce the present extent of HEC. Such programs should be integrated with conservation awareness camps for the local communities regarding spatial, seasonal and temporal patterns of crop-raids, human drunkenness and impact on HEC. Village elders and community leaders should also discourage human drunkenness and provocative behavior such as harassing or chasing elephants within their respective localities. Solar and electric fences can be set up around crop fields, human settlements (Hedges & Gunaryadi, 2010; Davies et al., 2011; Wijayagunawardane et al., 2016) and their effectiveness to deter elephants should be evaluated within such areas. Traditional crop guarding measures should be integrated with early warning systems (seismic and motion sensor triggered proximity alarms) and beehive fencing around identified hotspots (Fernando et al., 2008; King et al., 2017). Flash lights should be put up around crop fields, farmers can be provided with torchlights and fences can be covered with chili-oil socked rags ( Hoare, 2015; Gunaryadi & Sugiyo, 2017). Villagers can also be trained to prepare chili powdered bombs and use guard dogs to deter elephants near settlements ( Hoare, 2015). Unpalatable yet economically beneficial crops such as ginger, garlic, chillies, lemongrass should be grown in fields regularly visited by elephants (Gross et al., 2017). Such cash crops could act as deterrents as well as provide income for the local communities (Fernando et al., 2008). Timely compensation of crop damage incidents should also be provided as such measures will improve societal tolerance towards elephants (Gross et al., 2017). Small-scale community based tourism initiatives should also be explored within the hotspots to reduce extensive crop cultivation and generate economic benefits from wildlife (Ogutu, 2002). Radio-telemetry studies should be undertaken to understand the activity and resource utilization patterns of elephants at the interface between protected areas and the surrounding human-dominated landscape (Venkataraman et al., 2005; Buchholtz et al., 2019).

## Conclusion

Our study helps to untangle the relations between crop depredation, cropping pattern, land use type and human behavior, activity within a multi-use landscape of South Asia. We recommend further research on quantification of property damage, evaluation and comparison of multiple (long and short term) mitigation measures, age and gender specific elephant movement behavior. Studies should also be undertaken to understand the effect of crop fields, fragmentation and human presence on nocturnal habitat utilization by elephants. Long term monitoring of the HEC hotspots should be carried out to examine any changes in seasonal, temporal patterns of crop raids.

HEC remains a serious conservation challenge for managers, conservationists in Asia and Africa threating safety, livelihoods of rural communities and survival of elephant populations. Considering the limitations to animal dispersal, gene flow, and financial investments in fencing protected reserves, current strategies to physically separate elephant and humans as is done in parts of southern Africa cannot be advocated for rest of the elephant populations. Moreover, size of protected areas is comparatively smaller in Asia than Africa. Efforts should be prioritized to monitor HEC hotspots, maintain connectivity between populations, invest in HEC mitigation measures and provide economic incentives to local communities for coexistence. With three-fourth of the present Asian elephant habitat fragmented as a result of anthropogenic impacts, future of Asian elephants depends on habitat improvement and reduction in HEC within larger heterogeneous landscapes.

##  Supplemental Information

10.7717/peerj.9399/supp-1 Data S1 Raw data on crop depredation by Asian elephants

10.7717/peerj.9399/supp-2 Data S2 Data on spatial predictors of HEC (5 sq.km)

10.7717/peerj.9399/supp-3 Table S1 List of all variables considered for spatial risk mapping of HEC List of variables used for HEC risk prediction

10.7717/peerj.9399/supp-4 File S1 Details of the analytical procedure for HEC risk mapping

10.7717/peerj.9399/supp-5 Appendix S1 Questionnaire sheet used for recording data on crop depredation by elephants

10.7717/peerj.9399/supp-6 Figure S1 Receiver operating curve (ROC) for 5 km^2^ Maxent models ROC curve indicates predictive power of the spatial model for crop depredation by elephants in North Bengal

## Additional Information and Declarations

### Field Study Permissions

The following information was supplied relating to field study approvals (i.e., approving body and any reference numbers): The Office of the Principal Chief Conservator of Forests, Wildlife & Chief Wildlife Warden, West Bengal of the Government of West Bengal Directorate of Forests approved this field study (Ref: WII/NMHS/HWC/2016; Memo No.: 5662/WL/4R-6/2016).

### Data Availability

The following information was supplied regarding data availability: The raw data and a questionnaire are available in the Supplemental Files.
