# Supplementary material for: Elephants in the neighborhood: patterns of crop-raiding by Asian elephants within a fragmented landscape of Eastern India
Source: PeerJ. 2020 Jul 2;8:e9399. doi: 10.7717/peerj.9399 (PMC7335499; doi:10.7717/peerj.9399)
Supplement: Table S1 — List of variables used for HEC risk prediction [file peerj-08-9399-s003.docx]

Table S1. List of all variables considered for spatial risk mapping of human-elephant conflicts

| **Serial No** | **Variable Type** | **Predictor Variables** | **Reference** | **Unit** |
| --- | --- | --- | --- | --- |
| 1. | **Land use type** | Euclidean distance to protected areas | Chen et al., 2016 | Meter |
| 2. |  | Area of forest | Wilson et al., 2013 | Square meter |
| 3. |  | Area of agriculture | Wilson et al., 2013 | Square meter |
| 4. |  | Area of tea plantation | Wilson et al., 2013 | Square meter |
| 5. |  | Area of riverine patches | Naha et al., 2019 | Square meter |
| 6. |  | Area of sand bed | Naha et al., 2019 | Square meter |
| 7. | **Anthropogenic** | Length of roads | Naha et al., 2019 | Kilometer |
| 8. |  | Human density | Sitati et al., 2003 | Unit per km |
| 9. |  | Area of human settlements | Naha et al., 2019 | Square meter |
| 10. | **Water** | Length of rivers | Naha et al., 2019 | Kilometer |
