# Supplementary material for: Elephants in the neighborhood: patterns of crop-raiding by Asian elephants within a fragmented landscape of Eastern India
Source: PeerJ. 2020 Jul 2;8:e9399. doi: 10.7717/peerj.9399 (PMC7335499; doi:10.7717/peerj.9399)
Supplement: File S1 [file peerj-08-9399-s004.docx]

**Details of the analytical procedure for HEC risk mapping**

The elephant crop depredation risk model was determined from a set of environmental layers for a set of grid cells in the landscape, together with the crop raid locations. The model calculated the suitability of each grid cell as a function of the environmental/predictor variables within each cell.  A high value of the function at a particular grid cell indicated that the cell was predicted to have suitable conditions for crop depredation. The final computed model was a probability distribution over all the grid cells

To run MAXENT MODEL, we downloaded and installed the Maximum Entropy Species Distribution Modeling application along with Java script. After the applications was installed, we downloaded and digitized the environmental/predictor layers through various open sources

We superimposed 25 (5*5) and 5 km² grids on the study area. This was done with the help of ArcGIS 10.2.2. For 25 km² grids, the polygon width and height was 5*5 and consisted of 600 grids and for 5 km² grids it was 2.236 and consisted of 2780 grids in total. After the grids were created over the study area we clipped all predictor variables to 5 and 25 km² grid size. Then we extracted information for different land use types and anthropogenic variables in ArcGIS. We calculated Euclidean distance of each grid from the protected area layer. We tabulated area of each land use type using the supervised imagery of North Bengal. Once all vector files were prepared we converted them to raster files using the predefined specifications. After all rater files were ready, we converted them to ASCII files because this was the file extension specified by MAXENT ENTROPY MODEL.

We prepared a .csv file of crop raiding locations and defined the necessary projection

Then we added the .csv file and all other predictor variables (ASCII files). We specified (create response curves) and do jackknife to measure variable importance. We chose Logistic as a model output. Additional changes that we did were specifying random seed. We also increased the random test to 50 as this setting allowed us to withhold a certain percentage of the presence data to be used to evaluate the model performance. This is important because without these test data, the model will employ data used to develop the model (also called training data) to evaluate the model. This is a bias method and will provide an inflated measure of model performance. We specified the number of replicates to 5 which allowed the model to run multiple times and then we conveniently averaged the results from all the models created. Using this feature in combination with withholding a certain portion of the data for testing enabled us to evaluate the model performance while taking advantage of all available data without having an independent dataset. Executing multiple runs also provided a way to measure the amount of variability in the model. We changed the replicated run type from crossvalidate to subsample Further we used 500 iterations which allowed the model to have adequate time for convergence. The final output showed different response curves for all the independent predictors along with their averaged contribution, permutation importance.
