# Supplementary material for: Elephants in the neighborhood: patterns of crop-raiding by Asian elephants within a fragmented landscape of Eastern India
Source: PeerJ. 2020 Jul 2;8:e9399. doi: 10.7717/peerj.9399 (PMC7335499; doi:10.7717/peerj.9399)
Supplement: Appendix S1 [file peerj-08-9399-s005.docx]

**DATA SHEET FOR HOUSE DAMAGE & CROP DEPREDATION BY ASIAN ELEPHANT**

**Forest division: Block:**

**Locality/Village: Date:**

| **Gps loc (Lat, Long)** | **Altitude** | **Probable time of raid/ Date/ season / year** | **Species of crop raided** | **Condition of house damaged (Concrete/Wooden/Bamboo/Mud)** | **Number of elephants involved (herd size/loner)** | **Presence of stored granaries in village** | **Age and sex of raiding elephant** | **Major activity of humans in village or crop field** | **Number of humans in crop field or house** | **Time spent by elephant in raid/damage** | **Availability of rice beer/haaria in village** |
| --- | --- | --- | --- | --- | --- | --- | --- | --- | --- | --- | --- |
|  |  |  |  |  |  |  |  |  |  |  |  |
|  |  |  |  |  |  |  |  |  |  |  |  |
|  |  |  |  |  |  |  |  |  |  |  |  |
|  |  |  |  |  |  |  |  |  |  |  |  |
|  |  |  |  |  |  |  |  |  |  |  |  |
|  |  |  |  |  |  |  |  |  |  |  |  |
|  |  |  |  |  |  |  |  |  |  |  |  |
|  |  |  |  |  |  |  |  |  |  |  |  |
