# Supplementary figures and images for: Elephants in the neighborhood: patterns of crop-raiding by Asian elephants within a fragmented landscape of Eastern India
Source: PeerJ. 2020 Jul 2;8:e9399. doi: 10.7717/peerj.9399 (PMC7335499; doi:10.7717/peerj.9399)

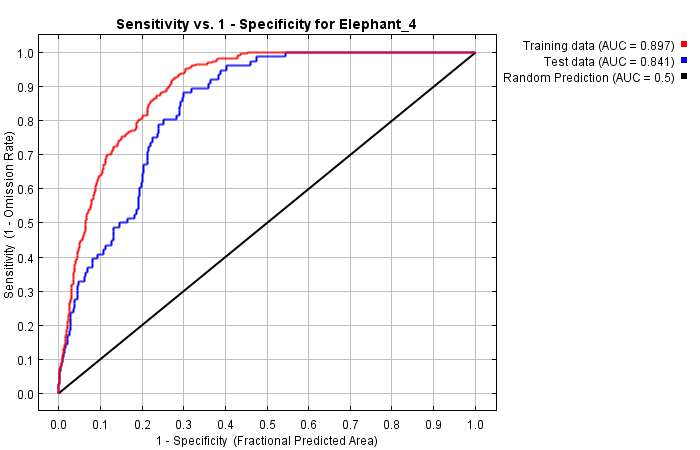

Supplement: Figure S1 — ROC curve indicates predictive power of the spatial model for crop depredation by elephants in North Bengal [file peerj-08-9399-s006.png]
